# Degradation Prediction of PEMFCs Based on Discrete Wavelet Transform and Decoupled Echo State Network

**DOI:** 10.3390/s25072174

**Published:** 2025-03-29

**Authors:** Jie Sun, Wenshuo Li, Mengying He, Shiyuan Pan, Zhiguang Hua, Dongdong Zhao, Lei Gong, Tianyi Lan

**Affiliations:** 1 School of Electrical and Control Engineering, Shaanxi University of Science and Technology, Xi’an 710021, China; jiesun_2025@163.com (J.S.); gong_lei@sust.edu.cn (L.G.); 4660@sust.edu.cn (T.L.); 2 School of Environment and Safety Engineering, North University of China, Taiyuan 030051, China; s202314011@st.nuc.edu.cn; 3 Xi’an Institute of Microelectronics, China Aerospace Industry Corp., Xi’an 710065, China; 89hmy@163.com; 4 School of Automation, Northwestern Polytechnical University, Xi’an 710072, China; panshiyuan@mail.nwpu.edu.cn

**Keywords:** fuel cell, degradation prediction, echo state network, discrete wavelet transform, remaining useful life

## Abstract

Predicting the degradation process of proton exchange membrane fuel cells (PEMFCs) under diverse operational conditions is crucial for their maintenance planning and health monitoring, but it is also quite complex. The variability in dynamic conditions and the shortcomings of short-term forecasting methods make accurate predictions difficult in practice. To strengthen the precision of deterioration predictive methods, this study introduces a degradation prediction of PEMFCs incorporating discrete wavelet transform (DWT) and a decoupled echo state network (DESN). The high-frequency noise is shielded by wavelet decomposition. Within data-driven approaches, an echo state network (ESN) can estimate the decline in PEMFC performance. To address the issue of low forecasting precision, this paper introduces a novel DESN with a lateral inhibition based on the decreasing inhibition (DESN-Z) mechanism. This enhancement aims to refine the ESN structure by mitigating the impact of other neurons and sub-reservoirs on the currently active ones, achieving initial decoupling. The lateral inhibition mechanism expedites the network’s acquisition of pertinent information and refines predictions by intensifying the rivalry among active neurons while suppressing others, thereby diminishing neuron interconnectivity and curbing redundant internal state data. Overall, combining DWT with DESN-Z (DDESN-Z) bolsters feature representation, promotes sparsity, mitigates overfitting risks, and enhances the network’s generalization capabilities. It has been demonstrated that DDESN-Z significantly elevates the precision of long-term PEMFC degradation predictions across static, quasi-dynamic, and fully dynamic scenarios.

## 1. Introduction

Amid the intensifying international energy crunch and growing sustainability concerns, the industrial sector has zeroed in on the pursuit of clean energy technologies as a key area of research [1]. Proton exchange membrane fuel cells (PEMFCs) have emerged as a paradigm of eco-friendly and efficient energy conversion devices, offering tremendous potential for use in the electric vehicle industry [2,3]. However, PEMFCs face challenges in practical applications, notably high costs and inadequate durability, which hinder their commercial feasibility and market penetration [4,5]. Therefore, it is crucial to thoroughly investigate PEMFC degradation mechanisms and develop reliable methods for predicting their performance decline and lifespan [6].

The primary approaches for prognosticating the remaining useful life (RUL) encompass model-based, data-oriented, and integrated methodologies [7,8]. Model-based strategies delve into degradation processes using physicochemical principles or mechanisms [9], requiring minimal data but posing significant challenges in model development [10]. He et al. [11] developed an empirical model to analyze PEMFC performance degradation across various conditions. Additionally, in [12], a model-based method was introduced and tested for robustness and validity under uncertain operational conditions. The hybrid method included the synergy between data-oriented methods, as well as the integration of model-based and data-oriented methods [13]. The pairing calculation of the two data-driven methods was complex and lacked high interpretability. The integration of model-driven and data-driven methods [14,15] faces the same problem as model-driven methods [16]. Data-driven methods do not necessitate prior knowledge and only obtain a suitable model through training data [17]. In [18], the authors present a practical approach leveraging the Fréchet distance and an extreme learning machine optimized by particle swarm optimization for precise predictions. A method integrating the Bayesian gated recurrent unit paired with convolutional neural network–long short-term memory, estimation, and uncertainty quantification was proposed in [19].

The echo state network (ESN), introduced by Jaeger in 2001, is an innovative recurrent neural network distinguished by its sparse connectivity, weight sharing, and robust long-term memory capabilities. These features render it highly suitable for addressing sequence data and time-dependent challenges. Notably, ESN circumvents the gradient disappearance or explosion issues common in traditional recurrent neural networks (RNNs) [20]. Across various domains, including image recognition, target tracking, text classification, sentiment analysis, and the optimal control of complex systems, ESN has demonstrated widespread applicability and notable achievements, particularly in time series prediction. In the realm of fuel cells, ESN was initially applied to fault diagnosis and subsequently yielded promising results in degradation prediction. He et al. [21] suggested combining the least absolute shrinkage and selection operator with ESN for improved performance. In [22], they introduced an ESN cycle reservoir with a jump (CRJ) to enhance prediction accuracy. In [23], the researchers proposed a multi-reservoir ESN for voltage degradation forecasting. Morando et al. [24] utilized ESN for voltage aging prediction and conducted a variance analysis to examine the impact of different ESN parameters. Li et al. [25] proposed a method for forecasting performance deterioration in PEMFCs, based on a bidirectional long short-term memory (Bi-LSTM)–gated cycle unit (GRU)–ESN fusion prediction framework. By combining the deep learning capabilities of Bi-LSTM and GRU, as well as the dynamic system modeling advantages of ESN, this method effectively captured the complex dynamic characteristics of PEMFC degradation and significantly improved the predictive precision. Gibey et al. [26] proposed a diagnosis and prediction method under an industrial framework to guide preventive maintenance and system control.

With the goal of improving the forecast accuracy of PEMFC multi-time scale degradation, a decreasing suppression decoupled echo state network based on discrete wavelet transform (DWT) and particle swarm optimization (PSO) is proposed. Firstly, the degradation voltage is used to characterize the aging performance. The multi-time scale degradation index is decomposed by wavelet decomposition, and the low-frequency and high-frequency signals are extracted, respectively. Then, a new ESN structure is constructed, the network sub-reservoir structure is improved to obtain a decoupled ESN, and the decreasing inhibition mechanism is added to obtain DESN-Z. The low-frequency signal that retains the original degradation trend is used as input, and the long-term prediction is performed after training by the DESN-Z network. Finally, the DWT-DESN-Z (DDESN-Z) network structure parameters are optimized by PSO, which shows that the exactness of the long-term projections for PEMFC’RUL is improved. The essential contributions of this paper are as follows:

(1) The degradation of the PEMFC is separated into a low-frequency signal that is shielded and a high-frequency signal that is retained to obtain smooth degradation curve characterization.
(2) A method that involves a decoupled echo state network with a mechanism for decreasing inhibition is proposed. This approach further decouples the network structure to enable accurate prediction.
(3) The degradation is precisely predicted by optimizing parameters using PSO, ensuring that the structural and weight superposition parameters of the sub-reservoir are optimal.

In Section 2, the operating test and implementation plan are introduced. A novel prediction method in proposed in Section 3. The test results are shown in Section 4. Section 5 presents the main conclusions.

## 2. Experimental Platform and Implementation Plan

### 2.1. Experimental Platform

**Figure 1:**
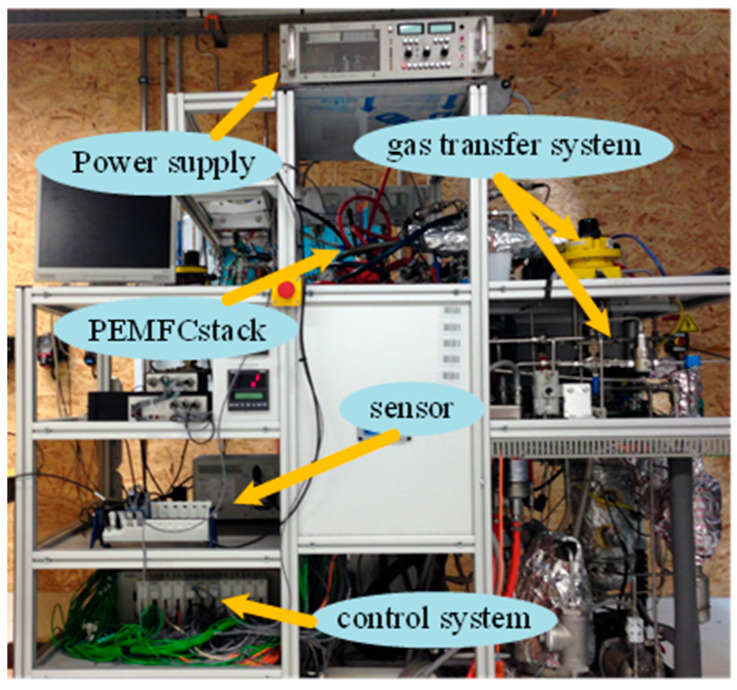
The PEMFC system’s experimental setup.

The dataset from the Fuel Cell Laboratory (FCLAB) [27], which comprises aging data under varying current conditions, is employed to validate the life prediction methodology. The operating state and parameter configuration of the PEMFC system under static (FC1), quasi-dynamic (FC2), and fully dynamic conditions (FC3) are recorded. In this study, the terminal voltage serves as a key condition metric, and the enhanced model undergoes multi-step predictions using datasets collected under diverse operational scenarios. The experimental setup of the PEMFC system is presented in Figure 1. A selection of working parameters is presented in Table 1.

An adaptive filter, leveraging the least mean square (LMS) algorithm, is utilized to filter and reconstruct the dataset. This process aims to preserve the degradation trend of the original output voltage while eliminating significant interference noise signals. Following wavelet decomposition, the refined network structure parameters are configured as specified: The input weight ***W***_in_ ranges between −0.5 and 0.5, the sub-reservoir cycle weight ***W*** falls within [−0.5, 0.5], and the sub-reservoir neurons are set as equal (*N*1 = *N*2 = *N*3 = *N*4). Additionally, the leakage rates *α*1, *α*2, *α*3, and *α*4 lie between 0 and 1 (exclusive), the output regularization factors *γ*1, *γ*2, *γ*3, and *γ*4 are within the interval (0.001, 0.009], and the parallel output combined weights *p*1, *p*2, and *p*3 are within (0.01, 0.99].

### 2.2. Implementation Plan

**Figure 2:**
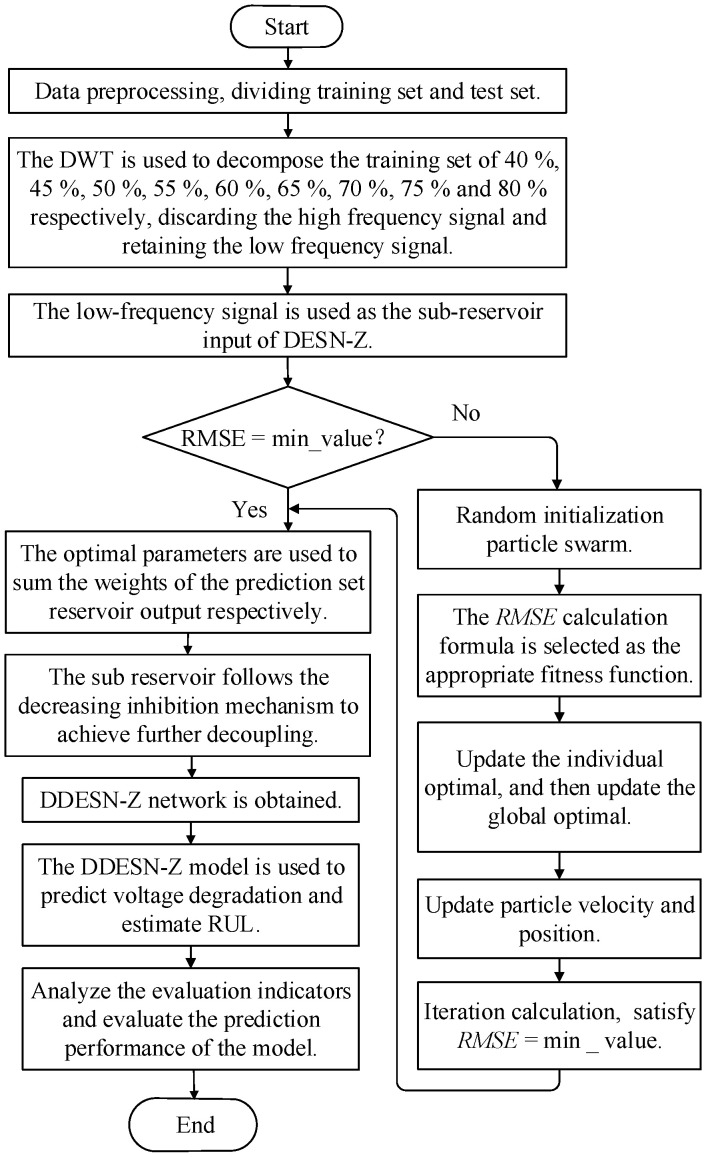
The implementation scheme for constructing DDESN-Z.

This study combines DWT and DESN-Z and uses PSO to optimize structural parameters to achieve the accurate prediction of PEMFC performance. Specifically, the aging process of PEMFCs is divided into low-frequency and high-frequency signals by DWT, and the low-frequency signal is retained to characterize the aging process. Then, corresponding to the input interface of DESN-Z, the low-frequency result of the final decomposition is selected as the different inputs of DESN-Z. Finally, the PSO is leveraged to optimize the parameters of DDESN-Z to achieve an accurate estimation of PEMFC wear-out and RUL. The implementation scheme for constructing DDESN-Z is shown in Figure 2.

### 2.3. Evaluation Index

This study employs the output voltage as a key metric. Following dataset filtration and reconstruction across the various operating conditions, the original output voltage’s substantial noise is mitigated while preserving its degradation pattern. The root mean square error (RMSE) and mean absolute percentage error (MAPE) serve as the benchmarks for evaluating performance, with their respective calculation formulae detailed subsequently.

(1) $$\mathrm{RMSE}=\sqrt{\frac{1}{M}\overset{M}{\underset{m=1}{\sum }}{\left(t_{l}\left(k\right)-t^{\prime }\left(k\right)\right)}^{2}}$$

(2) $$\mathrm{MAPE}=\frac{1}{M}\overset{M}{\underset{m=1}{\sum }}\left|\frac{t_{m}\left(k\right)-t^{\prime }\left(k\right)}{t_{m}\left(k\right)}\right|$$

where *t*(*k*) is the predicted voltage, *t*′(*k*) denotes the actual voltage, and *M* corresponds to the count of predicted outcomes.

## 3. Mathematical Model

### 3.1. Discrete Wavelet Transform

Fourier analysis has found extensive application in the realm of time-frequency domain conversion, enabling the breakdown of an original signal into sine waves varying in frequency. However, this process entails the loss of temporal information. To address this limitation of traditional Fourier analysis, the wavelet transform (WT) has been introduced. By employing dilation and translation, WT can examine local characteristics across various time and frequency scales [28]. In WT, extended periods are assigned to the lower-frequency components of the signal, whereas shorter intervals are used for high-frequency components. Consequently, WT excels in examining nonstationary signals and has been adopted in the diagnostics of systems [29]. The serial form of the wavelet transform is denoted as follows:

(3) $$C(a,\;b)=\frac{1}{\sqrt{a}}\int_{-\infty }^{+\infty }f(k)\psi^{*}(\frac{k-b}{a})dk$$

where *f*(*k*) represents the initial signal, with *ψ*(*k*) being the primary wavelet and *ψ*^∗^(*k*) its conjugate of a complex number. The wavelet dilation and translation factors are denoted as *a* (*a* > 0) and *b* (*b* ∈ ***R***), respectively. We utilize the Daubechies (DB4) wavelet due to its capacity to redistribute signal energy, focusing most of it on the estimated part. A smaller *a* compresses the wavelet, while a larger value of *a* stretches it. For discretization, we typically use a scale factor of 2^p^q. The discrete wavelet transform (DWT) is then defined based on these parameters and the chosen wavelet function.

(4) $$D(p,\;q)=2^{-p/2}\int_{-\infty }^{+\infty }f(k)\psi^{*}(2^{-p}k-q)dk$$

where *p* and *q* are both whole numbers, the DWT of the initial signal is determined utilizing the Mallat algorithm [25].

(5) $$\left\{\begin{array}{c} S_{2^{p}}f(k)=\sum_{q}h_{q}S_{2^{p-1}}f(k-2^{-p}q)\\ W_{2^{p}}f(k)=\sum_{q}g_{q}S_{2^{p-1}}f(k-2^{-p}q) \end{array}\right.$$

where *S*_2*p*_ is the smoothing operator with *S*_0_*f*(*k*) = *f*(*k*). *W*_2*p*_*f*(k) represents the DWT of *f*(*k*), while *h_q_* and *g_q_* are parameters of the orthogonal filtering system.

(6) $$\left\{\begin{array}{c} H_{low}(\omega )=\sum_{q}h_{q}e^{-iq\omega }\\ G_{high}(\omega )=\sum_{q}g_{q}e^{-iq\omega } \end{array}\right.$$

Subsequently, the signal *f*(*k*) denotes the following:

(7) $$\begin{array}{l} f(k)=\sum_{q}cA_{0}\psi_{p,q}(k)\\ =\sum_{q}cA_{1}\psi_{p-1,q}(k)+\sum_{q}cD_{1}\psi_{p-1,q}(k)\\ =cA_{1}(k)+cD_{1}(k) \end{array}$$

Using the method of signal decomposition at multiple resolutions, at each level of decomposition, m (from 0 to *M*) of the signal breaks down into an approximation component (*cA_m_*_+1_), capturing features at low frequencies or broader scales, and a detail component (*cD_m_*_+1_), capturing features at high frequencies or finer scales of *cA_m_*. Consequently, *f*(*k*) is disintegrated into *M* such layers.

(8) $$\begin{array}{l} f(k)=cA_{1}(k)+cD_{1}(k)\\ =cA_{2}(k)+cD_{2}(k)+cD_{1}(k)\\ =cA_{M}(k)+\sum_{m=1}^{M}cD_{m}(k) \end{array}$$

Basically, predicting the lifespan of a PEMFC system involves managing time-varying series data. The DWT process of breaking down is advantageous for addressing dynamic health indicators that exhibit properties across various temporal scales.

### 3.2. Echo State Network

**Figure 3:**
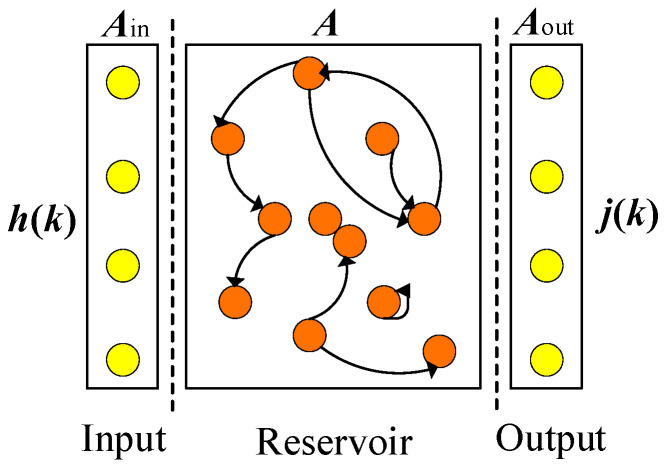
The classical network structure of ESN.

An ESN is an innovative neural network designed for processing time-series data. It simulates the “internal state” of the network by connecting a vast number of neurons within a reservoir. This network comprises input units, a reservoir, and output units, and it fine-tunes parameters such as leakage rate, spectral radius, and regularization to determine the output weights, as outlined in [30]. The classical network architecture of the ESN is depicted in Figure 3.

### 3.3. Decoupled Echo State Network

**Figure 4:**
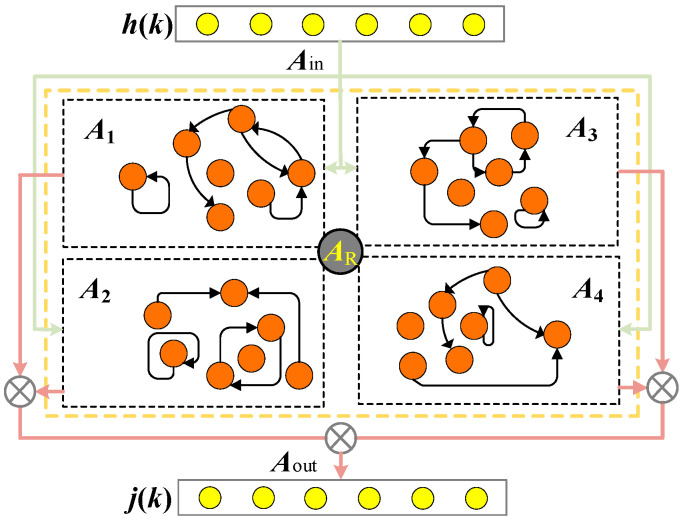
The network structure of DESN.

Drawing inspiration from the ESN, the DESN was developed, featuring multiple sub-reservoirs, each potentially containing an equal or unequal number of neurons. These sub-reservoirs are tasked with capturing diverse temporal patterns or features within the input data, thereby augmenting the network’s flexibility and complexity, as noted in [31]. The DESN accomplishes decoupling by modulating the connection weights among the various sub-reservoirs, facilitating independent learning and adaptation to distinct features. The DESN configuration is presented in Figure 4.

### 3.4. Decoupled Echo State Network with Z-Scheme

**Figure 5:**
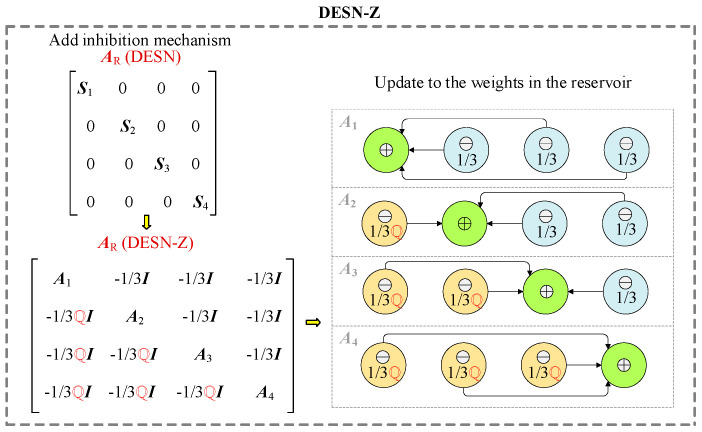
The network structure of DESN-Z.

In our study, we introduce a reservoir-decreasing inhibition-based inhibition mechanism (Z-scheme) into the DESN to bolster decoupling and minimize extraneous information. The structure (DESN-Z) fosters competition among active neurons while suppressing others, thereby expediting the acquisition of valuable information within the network and further refining prediction accuracy. The network structure of DESN-Z can be seen in Figure 5.

In the DESN-Z framework, the quantities of nodes designated for the input, state, and output of the sub-reservoir are *L*, *M*, and *N*, respectively. Utilizing the parameter vector ***G****_i_*, we assign it as the input ***h***(*k*) for the DESN-Z. The formula for updating the corresponding state of the sub-reservoir is as follows:

(9) $$\tilde{v}\left(k\right)=f\left(A_{\mathrm{in}}*h\left(k\right)+A*v\left(k-1\right)\right)$$

(10) $$v\left(k\right)=\left(1-\alpha \right)*v\left(k-1\right)+\alpha *\tilde{v}\left(k\right)$$

At time step *k*, ***v***(*k*) ∈ ℝ^(*NM*)×1^ and $\tilde{v}(k)$ ∈ ℝ^(NM)×1^, respectively, denote the activation vector and its corresponding updated state of the neurons within the reservoir. The neurons’ activation function is represented as *f*(·), which is typically the tanh(·) function. ***A****_in_* ∈ ℝ^(*NM*)×(*NL*)^ is the input weight value matrix, and ***A*** ∈ ℝ^(*NM*)×(*NM*)^ represents the feedback weight matrix inside the reservoir. The input vector at time (*k* − 1) is denoted as ***h***(*k* − 1) ∈ ℝ^(*NL*)×1^. The leakage rate of the reservoir is given by *α*, which lies within the interval (0,1]. The equation governing the output state is expressed as follows:

(11) $$j\left(k\right)=A_{\mathrm{out}}*\left[h\left(k-1\right);v\left(k\right)\right]$$

where ***j***(*t*) ∈ ℝ^(*NN*)×1^ serves as the output weight matrix. By employing ridge regression, the root mean square error (RMSE) is computed and, subsequently, the output weight matrix ***A****_out_* is determined.

(12) $$A_{\mathrm{out}}=arg\;min\sqrt{\frac{1}{N}\overset{N}{\underset{n=1}{\sum }}{\left(j_{n}\left(k\right)-j_{n}{\left(k\right)}^{\mathrm{target}}\right)}^{2}}$$

(13) $$A_{\mathrm{out}}=G^{\mathrm{target}}U^{T}{\left(UU^{T}+\gamma I\right)}^{-1}$$

In this context, *N* signifies the total count of data points within the training datasets. ***U*** represents the reservoir’s output matrix, while ***G****^target^* denotes the matrix of target output values. *γ* functions as a regularization coefficient, and ***I*** denotes the identity matrix. To enhance the reservoir, a lateral inhibition hiding mechanism is incorporated. This results in the determination of the reservoir’s recurrent weights and the updated states of the four sub-reservoirs are derived as follows:

(14) $$A_{R}=\left[\begin{array}{cccc} A_{1} & -\frac{1}{3}I & -\frac{1}{3}I & -\frac{1}{3}I\\ -\frac{\mathbb{Q}}{3}I & A_{2} & -\frac{1}{3}I & -\frac{1}{3}I\\ -\frac{\mathbb{Q}}{3}I & -\frac{\mathbb{Q}}{3}I & A_{3} & -\frac{1}{3}I\\ -\frac{\mathbb{Q}}{3}I & -\frac{\mathbb{Q}}{3}I & -\frac{\mathbb{Q}}{3}I & A_{4} \end{array}\right]$$

where ***A****_R_* ∈ ℝ^(*NM*)×(*NM*)^ denotes the recurrent weight matrix of the DESN-Z reservoir, while ***I*** ∈ ℝ^(*NM*)/4×(*NM*)/4^ represents the identity matrix. Within this matrix, ***A****_i_* ∈ ℝ^(*NM*)/4×(*N*M)/4^ (*i* = 1, 2, 3, 4) signifies the recurrent weight of each of the four sub-reservoirs. The equation that governs the corresponding updated state of the reservoir is formulated as follows:

(15) $$\tilde{v}\left(k\right)=f\left(A_{\mathrm{in}}*h_{\mathrm{whole}}\left(k\right)+A_{R}*v\left(k-1\right)\right)$$

(16) $$v\left(k\right)=\left(I-\alpha \right)*v\left(k-1\right)+\alpha *\tilde{v}\left(k\right)$$

The overall reservoir’s updated state is represented by a new matrix $\tilde{v}(k)$ ∈ℝ ^((*N*M)/4)×4^ at time *k*. Additionally, ***v***(*k*) ∈ ℝ^((*NM*)/4)×4^ is arranged to form a new input matrix ***h***_whole_(*k*) ∈ ℝ^(*NL*)×4^, with ***A****_in_*_(*i*)_ ∈ ℝ^((*NM*)/4)×(*NL*)^ combined to form ***v***(*k* − 1) ∈ ℝ^((*NM*)/4)×4^, which denotes the identity matrix, and ***α****_i_* ∈ ℝ^(*NM*)/4×(*NM*)/4^. For specific matrix operations, ℚ is introduced as a prediction operator, tasked with computing the correlation of the sub-reservoir state ℚ·***v****_i_*(*k* − 1) ∈ ℝ^((*NM*)/4)×1^ at time (*k* − 1).

(17) $$\mathbb{Q}\cdot v_{i}\left(k-1\right)=f(A_{\mathrm{in}(i)}*h\left(k\right)+A_{i}*v_{i}\left(k-1\right))$$

By substituting Equation (9) into (6), and incorporating Equation (8), we can compute ***A****_R_*. When combined with Equations (3)–(5), we obtain the output weight matrix for all sub-reservoirs, denoted as ***A****_out_* ∈ ℝ^((*NN*)×4)×(*NL*+(*NM*)/4)^. From the output weight of each sub-reservoir, ***A****_out_*(*i*) ∈ ℝ^(*NN*)×(*NL*+(*NM*)/4)^, we can derive four outputs ***h****_i_*(*k*) ∈ ℝ^(*NN*)×1^.

(18) $$j_{i}\left(k\right)=A_{\mathrm{out}(i)}*\left[h\left(k-1\right);v_{i}\left(k\right)\right]$$

Based on the output weights, the outputs of the sub-reservoirs are sequentially summed.

(19) $$j_{12}\left(k\right)=w_{1}*j_{1}\left(k\right)+(1-w_{1})*j_{2}\left(k\right)$$

(20) $$j_{34}\left(k\right)=w_{2}*j_{3}\left(k\right)+(1-w_{2})*j_{4}\left(k\right)$$

(21) $$j\left(k\right)=w_{3}*j_{12}\left(k\right)+(1-w_{3})*j_{34}\left(k\right)$$

where at time *k*, ***j***_12_(_k_) ∈ ℝ^(*NN*)×1^ represents the combined output vectors of the first and second sub-reservoirs, while ***j***_34_(*k*) ∈ ℝ^(*NN*)×1^ signifies the combined output vectors of the third and fourth sub-reservoirs. The vector ***j***(*k*) ∈ ℝ^(*NN*)×1^ is the summation of the output vectors ***j***_12_(*k*) and ***j***_34_(*k*), which serves as a prediction of the vector value in terms of the output voltage signal. Here, *w*_1_, *w*_2_ and *w*_3_ correspond to the sums of ***j***_1_(*k*) and ***j***_2_(*k*), ***j***_3_(*k*) and ***j***_4_(*k*), ***j***_12_(*k*) and ***j***_34_(*k*), respectively.

In the DESN-Z architecture, the parameters undergo optimization to enhance performance. The leakage rate *α_i_* serves as a metric for assessing the reservoir’s dynamic behavior, with smaller *α_i_* values exerting a more significant influence on predicting historical stack voltage data points. The spectral radius *ρ_i_* denotes the maximum eigenvalue magnitude of ***A****_i_* within the layered sub-reservoir structure. When the spectral radius is smaller than 1 under zero input conditions, it exhibits exceptional echo state properties, enabling the effective incorporation of multi-time scale characteristics of stack voltage inputs. By refining the reservoir structure and reconstructing the cyclic weight matrix ***A****_R_,* DESN-Z can more effectively extract features related to the aging degree of PEMFCs, thereby minimizing redundant information, mitigating overfitting risks, and augmenting the network’s generalization capabilities.

## 4. Result and Discussion

### 4.1. Under Steady Conditions (FC1)

**Figure 6:**
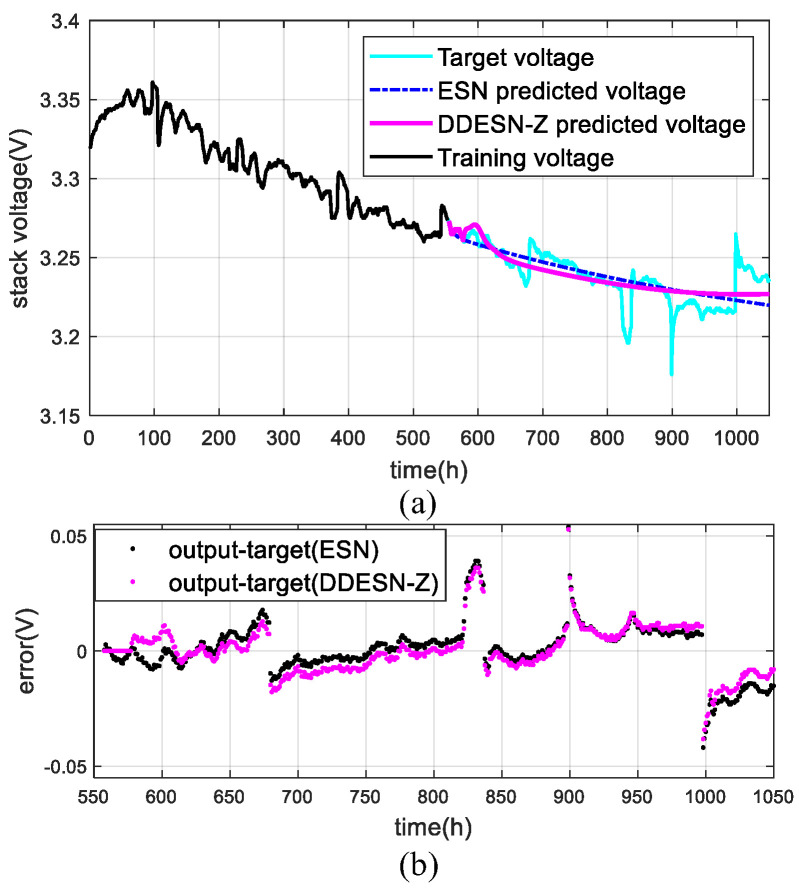
Results with DDESN-Z under steady conditions (FC1): (**a**) voltage; (**b**) error.

In a steady-state scenario, after preprocessing, the datasets totaling 1045 h are segmented into nine distinct groups. Each group is used for training with durations of 400 h, 450 h, 500 h, 550 h, 600 h, 650 h, 700 h, 750 h, and 800 h, respectively. The outcomes of utilizing the DDESN-Z model to predict output voltage are depicted in Figure 6.

The DDESN-Z model outperforms the traditional ESN in prediction accuracy. As shown in Figure 6, with a 550 h training period, DDESN-Z’s forecasted voltage trend closely matches the actual data. While the ESN predictions are linear, DDESN-Z shows a more curved pattern, aligning better with the expected voltage degradation. DDESN-Z also demonstrates superior performance, with its predicted voltages being closer to the target values than those of ESN.

Table 2 compares ESN and DDESN-Z predictions across various training durations. Notably, DDESN-Z shows reduced RMSE values of 6.64%, 17.59%, and 26.95% at 400 h, 650 h, and 800 h, and lower MAPE values of 0.01%, 7.97%, and 30.62%, respectively, compared to ESN. The DDESN-Z method enhances long-term voltage forecasting accuracy via multi-time scale inputs and reservoir decoupling.

### 4.2. Under Quasi-Dynamic Conditions (FC2)

**Figure 7:**
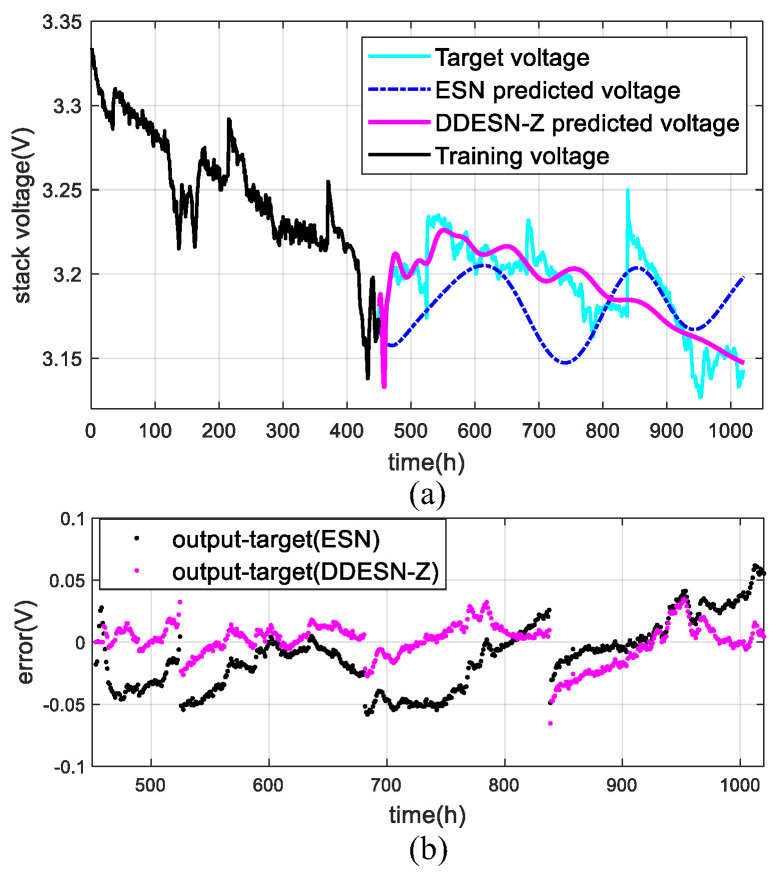
Results with DDESN-Z under quasi-varying conditions (FC2): (**a**) voltage; (**b**) error.

In a quasi-dynamic-state scenario, after preprocessing, the datasets totaling 1020 h are segmented into nine distinct groups. Each group is used for training with durations of 400 h, 450 h, 500 h, 550 h, 600 h, 650 h, 700 h, 750 h, and 800 h, respectively. The outcomes of utilizing the DDESN-Z model to predict output voltage are depicted in Figure 7.

The DDESN-Z model demonstrates superior predictive performance over the conventional ESN. As illustrated in Figure 7, DDESN-Z’s predictions align well with actual data across a 450 h training duration and notably excel in the 500 h to 750 h range. It also shows a smaller discrepancy between the predicted and target output voltages, indicating a more accurate prediction compared to ESN. On the one hand, the DESN-Z considering decoupling on the structure of the reservoir enables the improved precision of long-time prediction. On the other hand, DDESN-Z further utilizes DWT, which decomposes multi-time scale aging signals, preserving the trend signals that characterize the degradation of PEMFCs. During the training phase, DDESN-Z deeply learns complex information in nonlinear aging data. Therefore, in the prediction stage, the actual fluctuation situation can be better adapted, and the aging mechanism of PEMFC can be accurately predicted through the fitting trend of more fluctuations.

Table 3 displays the prediction outcomes for ESN and DDESN-Z across various training periods. Notably, DDESN-Z shows reduced RMSE values of 50.20%, 38.51%, and 42.21% at 450 h, 500 h, and 800 h, and lower MAPE values of 53.74%, 49.62%, and 41.04%, respectively, compared to the ESN. DDESN-Z enhances accuracy in long-term voltage forecasts by utilizing multi-time scale inputs and decoupling the reservoir.

### 4.3. Under Dynamic Conditions (FC3)

**Figure 8:**
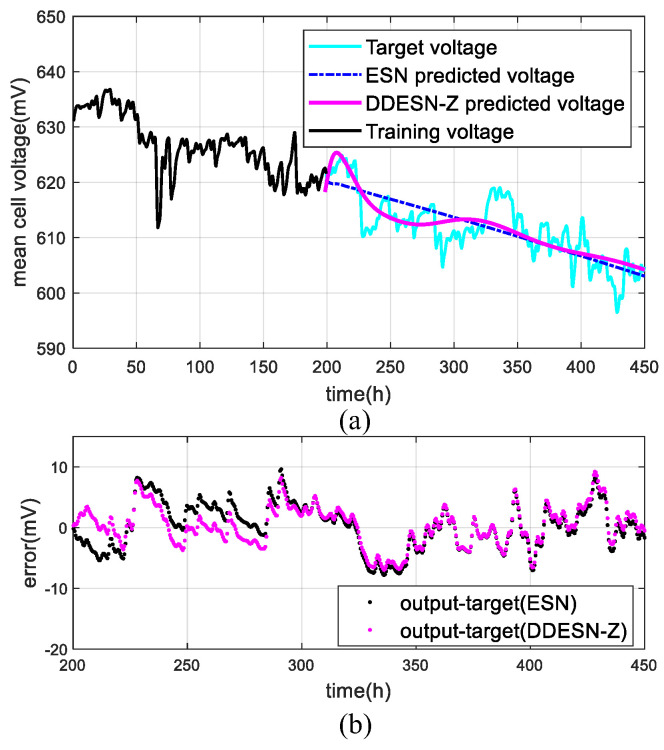
Results with DDESN-Z under quasi-dynamic conditions (FC3): (**a**) voltage; (**b**) error.

In dynamic scenarios, preprocessed datasets totaling 453 h are split into nine groups for training durations of 200 h to 400 h, incremented by 25 h each. The DDESN-Z model’s predictive performance for output voltage is presented in Figure 8. Notably, these voltage readings represent the average across 96 cells. To accommodate diverse loads, the fuel cell current was measured over cycles ranging from 20 A to 70 A and then to 100 A within 24 h. Table 4 presents the comparative forecasting outcomes of the ESN and DDESN-Z models across various training durations. Notably, DDESN-Z shows reduced values of 10.92%, 26.63%, and 29.29% at 200 h, 275 h, and 400 h, and lower MAPE values of 10.56%, 28.24%, and 28.80%, respectively, compared to the ESN. For extended voltage predictions, DDESN-Z enhances precision by incorporating multi-time scale inputs and implementing reservoir decoupling techniques.

Comparing the static (FC1) and quasi-dynamic (FC2) conditions, the prediction errors for FC3 are larger. There are two main reasons for this effect. The first reason is likely that the DWT filters the key features for FC3. However, it selects a reasonable decomposition level to avoid losing significant feature information of the original signal as much as possible. The second reason is that error accumulation can occur between the predicted trend and the actual frequent fluctuation signals in long-time forecasting. The results of a comparison between DESN-Z and DDESN-Z under different conditions are shown in Table 5.

The health indicator of a PEMFC is usually expressed by voltage or power. However, the complex aging mechanism inside a PEMFC is influenced by multiple factors. It cannot be ignored that the operating parameters of the stack are one of the factors affecting degradation. On the one hand, there is an inseparable relationship between the stack current and stack voltage. The output current of the stack is proportional to the consumption of the reactant gas. When the stack current increases, the consumption rate of the reactant gas increases. If the gas supply is insufficient or the performance of the gas diffusion layer decreases, the stack voltage will decrease. The aging parameters of the stack, such as proton exchange membrane resistance, catalyst activity, and gas diffusion layer performance, will affect the relationship between current and voltage. As the aging degree deepens, these parameters change, resulting in a change in the coupling relationship between current and voltage. On the other hand, the aging process of PEMFC is affected by the coupling relationship between various operating parameters and stack voltage, including temperature, humidity, and gas pressure. However, the coupling relationship between the parameters and the stack voltage is difficult to quantify, and further exploration is needed.

### 4.4. Estimation of Remaining Useful Life

**Figure 9:**
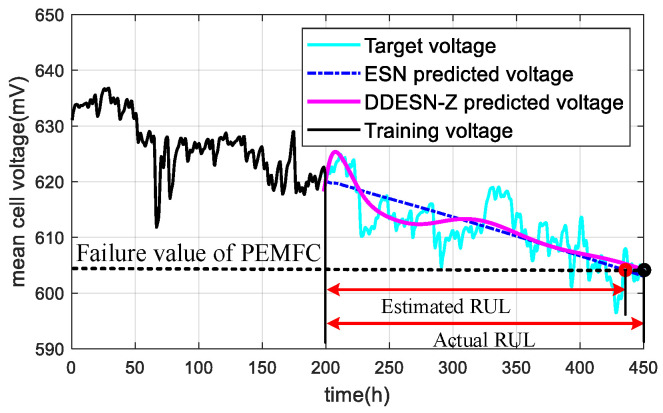
Computation of RUL.

The United States Department of Energy (U.S.DoE) states that users of PEMFC systems can define the appropriate failure thresholds for different application scenarios, such as vehicles, portable equipment, and stationary power plants. In this paper, the depletion time of a PEMFC is chosen as the end-of-life time. The RUL of a PEMFC was calculated by the difference between the termination time and the initial prediction time, as shown in Figure 9.

**Figure 10:**
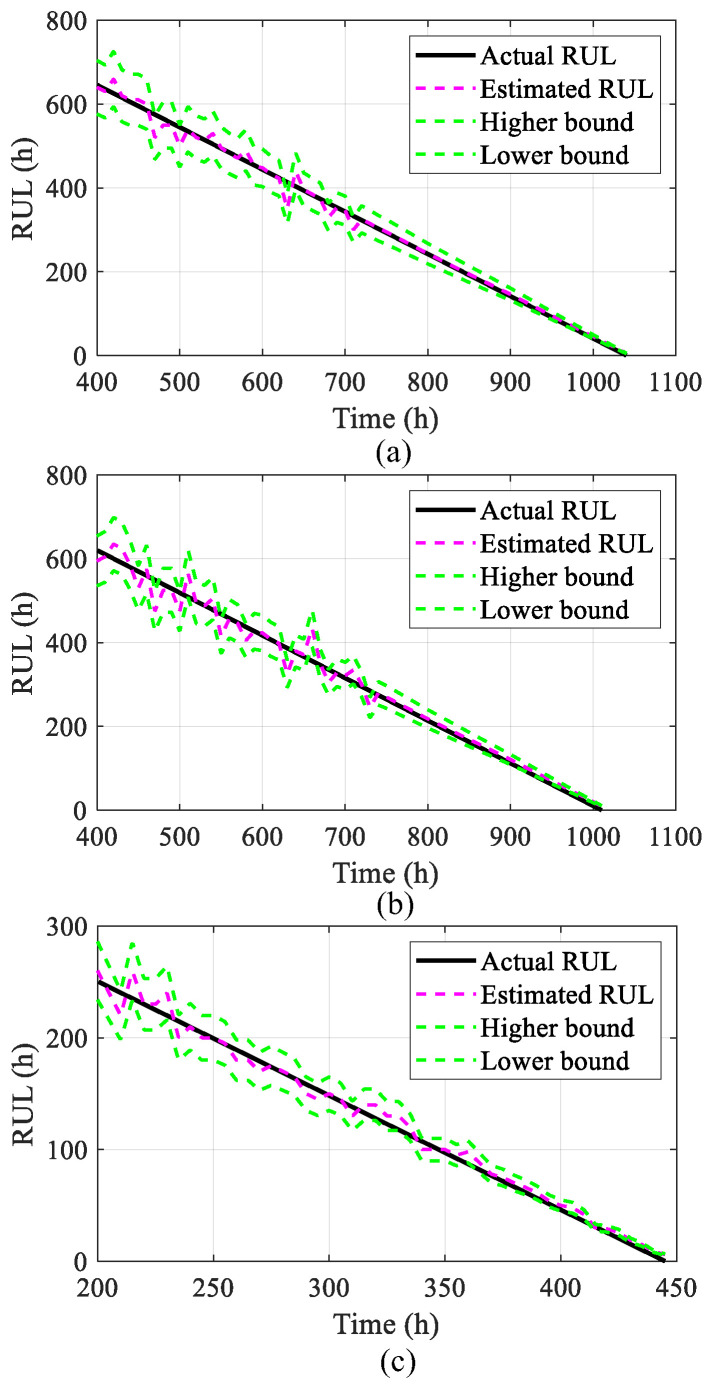
The estimated results of RUL: (**a**) under steady conditions, (**b**) under quasi-dynamic conditions, (**c**) under fully dynamic conditions.

This paper utilizes RMSE and MAPE as metrics to assess prediction accuracy, reflecting the discrepancy between the actual and forecasted values within the prediction range. Lower values of these indexes signify a more accurate voltage trend prediction. The network model determines when the estimated voltage intersects the failure tolerance level, from which the RUL of the PEMFC is deduced. The results of the RUL estimations are presented in Figure 10. Among them, a 95% confidence interval is set to estimate the RUL, forming the upper and lower envelope of the estimated RUL, as shown in Figure 10 as the higher boundary and lower boundary. By calculating the error margin as a percentage of the estimated RUL value, the upper and lower bounds of the confidence interval are obtained.

## 5. Conclusions

Accurate RUL prediction for PEMFCs relies on choosing the right degradation indicators and accurately anticipating the degradation trajectory. During PEMFC system degradation, an ESN can predict the voltage in multiple steps. By stacking reservoirs, the ESN model’s forecasting precision is boosted. However, there are multi-time scale problems in the internal aging of the system, while neuron and sub-reservoir coupling exists. Firstly, the high-frequency noise is shielded by wavelet decomposition, and the aging trend of the system is extracted. Then, to refine the ESN structure, the DESN initiates decoupling and then employs lateral inhibition to reduce neuron coupling and state redundancy. DDESN-Z strengthens feature representation, network sparsity, and generalization, speeding up useful information learning and enhancing predictions. This study applies DDESN-Z to PEMFC degradation prediction, confirming its low RMSE and MAPE values under varied conditions. In contrast to the model-driven approaches, DDESN-Z does not rely on complex and difficult-to-construct empirical and mechanistic models. Compared with other data-driven methods, such as RNN, LSTM, GRU, etc., DDESN-Z can improve the accuracy of long-term prediction of PEMFC aging by suppressing the coupling characteristics of the network structure. Compared with the classical ESN, DDESN-Z considers the multi-time scale aging of PEMFC while considering the coupling characteristics of the network structure, which further improves the prediction accuracy. Future research will delve deeper into the ESN structures for more precise PEMFC degradation forecasts. Looking ahead, research should focus on combining immediate estimation techniques with forecasting methods for the distant future. This integration will facilitate informed decision-making and enhance the resilience and flexibility of hybrid predictive models.

## Figures and Tables

**Table 1:** Parameters of the experimental platform.

Parameters	FC1	FC2	FC3
Number of single cells	5	5	96
Operating power (kW)	1	1	1~5
Activation area (cm^2^)	100	100	100
Operating current (A)	70	70 ± 7	20~99
Time (h)	1154	1020	450
Hydrogen input/output temperature (°C)	29/41	27/37	58
Air input/output temperature (°C)	42/51	43/51	58

**Table 2:** The results of FC1.

Training Time (FC1)	RMSE		MAPE	
	ESN	DDESN-Z	ESN	DDESN-Z
400 h	0.01039	0.00970	0.00216	0.00216
450 h	0.01050	0.01125	0.00229	0.00237
500 h	0.01098	0.01180	0.00243	0.00254
550 h	0.01128	0.01084	0.00244	0.00251
600 h	0.01207	0.01145	0.00264	0.00250
650 h	0.01228	0.01012	0.00276	0.00254
700 h	0.01161	0.01114	0.00270	0.00281
750 h	0.01222	0.01195	0.00301	0.00299
800 h	0.01228	0.00897	0.00307	0.00213

**Table 3:** Results of FC2.

Training Time (FC2)	RMSE		MAPE	
	ESN	DDESN-Z	ESN	DDESN-Z
400 h	0.01934	0.01745	0.00437	0.00407
450 h	0.02972	0.01480	0.00763	0.00353
500 h	0.02475	0.01522	0.00653	0.00329
550 h	0.02072	0.01591	0.00536	0.00353
600 h	0.01973	0.01574	0.00496	0.00359
650 h	0.019 66	0.017 14	0.004 83	0.004 13
700 h	0.01924	0.01640	0.00453	0.00397
750 h	0.01799	0.01750	0.00477	0.00458
800 h	0.01862	0.01076	0.00441	0.00260

**Table 4:** Results of FC3.

Training Time (FC3)	RMSE		MAPE	
	ESN	DDESN-Z	ESN	DDESN-Z
200 h	3.69178	3.28850	0.00483	0.00432
225 h	3.81518	3.51380	0.00509	0.00450
250 h	3.49377	3.25630	0.00468	0.00405
275 h	4.69151	3.44200	0.00609	0.00437
300 h	3.38791	2.64380	0.00435	0.00359
325 h	3.29354	2.40670	0.00432	0.00307
350 h	3.16738	2.42800	0.00431	0.00298
375 h	2.825 85	2.61900	0.00370	0.00332
400 h	2.89835	2.04930	0.00375	0.00267

**Table 5:** The results comparing DESN-Z with DDESN-Z under different conditions.

Training Time (h)		RMSE		MAPE	
		DESN-Z	DDESN-Z	DESN-Z	DDESN-Z
FC1	400	0.01001	0.00970	0.00216	0.00216
	600	0.01168	0.01145	0.00255	0.00250
	800	0.01108	0.00897	0.00278	0.00213
FC2	400	0.01849	0.01745	0.00430	0.00407
	500	0.02047	0.01522	0.00477	0.00329
	600	0.01732	0.01574	0.00394	0.00359
FC3	200	3.58188	3.28850	0.00469	0.00432
	300	3.38001	2.64380	0.00410	0.00359
	400	2.80106	2.04930	0.00353	0.00267

## Data Availability

This study’s raw sequence data are archived in the Fuel Cell Laboratory (FCLAB).
